# Kinetics of the serological response up to one year after tularemia

**DOI:** 10.3389/fcimb.2022.1072703

**Published:** 2023-01-06

**Authors:** Helena Lindgren, Johan Eklund, Kjell Eneslätt, Anders Sjöstedt

**Affiliations:** ^1^ Department of Clinical Microbiology, Umeå University, Umeå, Sweden; ^2^ Ljusdal-Ramsjö Primary Care Centre, Ljusdal, Sweden

**Keywords:** tularemia, serological response, kinetics, one year, elderly

## Abstract

Serological analysis is the predominant method used to diagnose tularemia, a zoonotic disease caused by the highly virulent bacterium *F. tularensis*. We determined *F. tularensis*-specific IgM and IgG antibody titers by an LPS-based ELISA assay on five occasions one to twelve months after onset of ulceroglandular tularemia in 19 individuals. Peak IgM antibody titers were observed at the one-month time point and peak IgG antibody titers at the two-month time point. Both IgG and IgM antibody levels declined linearly thereafter with rather similar kinetics. Compared to the average one-month antibody titers, average IgG titers were not significantly lower before the 12-month time point and IgM titers before the 4-month time point. All, but one average titer, were significantly increased compared to the cut-off of the assay. Average IgG and IgM titers were significantly lower for the group = 69 years old compared to the group < 69 years. Collectively, the data demonstrate a persistence of *F. tularensis*-specific IgM and IgG antibody titers for at least 12 months after ulceroglandular tularemia. Thus, low, but significantly elevated *F. tularensis*-specific antibody titers are of limited diagnostic value since they are not indicative of ongoing tularemia.

## Introduction

*Francisella tularensis* is the etiological agent of the zoonotic disease tularemia and it is a facultative intracellular, highly infectious, Gram-negative bacterium (Sjöstedt, 2007). Diagnosis of tularemia may be challenging since it is a disease rarely seen by most physicians and may therefore not be immediately recognized, and, in addition, some patients present with non-specific symptoms (Anda et al., 2007; Petersen et al., 2017; Maurin, 2020). Tularemia cases are almost exclusively reported from regions in the Northern Hemisphere and although many countries report few cases, there are regions where the disease is endemic. The highest total number of tularemia cases have been reported from European countries, most notably, Sweden, Finland, Turkey, Hungary and Czech Republic, but in these countries, as well as in other countries with tularemia, there are very marked annual and seasonal variations and most outbreaks are local with random occurrence but with a predominance during late summer and autumn (Tärnvik et al., 1996; Tärnvik et al., 2004; Hestvik et al., 2015; Rossow et al., 2015).

Tularemia demonstrates distinct clinical presentations, dependent on the route of infection and varies from localized symptoms, such as skin lesions and lymphadenopathy, to severe respiratory disease and potentially life-threatening invasive disease (Tärnvik and Berglund, 2003; Anda et al., 2007; Maurin, 2020). The clinical presentation is also dependent on the bacterial subspecies and subspecies *tularensis* (type A), if inhaled, causes a disease with high mortality if untreated, whereas the other clinically important subspecies, *holarctica*, very rarely causes fatal disease, although individuals may become severely ill, in particular due to the respiratory form (Tärnvik and Berglund, 2003; Anda et al., 2007; Petersen et al., 2017; Maurin, 2020).

The predominant forms of tularemia are the ulceroglandular and respiratory forms. The former normally depends on spread of bacteria by vectors, predominantly mosquitoes or ticks, and the latter form on inhalation of contaminated dust (Hestvik et al., 2015; Abdellahoum et al., 2020; Telford and Goethert, 2020). Irrespective of form, tularemia results in effective protective immunity, and only a few cases of reinfection have been reported (Tärnvik and Berglund, 2003; Kirimanjeswara et al., 2008; Conlan, 2011; Elkins et al., 2016). The long-lived protection is dependent on cell-mediated immunity, as for many other intracellular bacterial infections. In fact, evidence indicates that vigorous cell-mediated responses are detected for at least 25 years in almost all individuals after tularemia or tularemia vaccination (Ericsson et al., 1994; Ericsson et al., 2001; Eneslätt et al., 2011). In the case of tularemia, also humoral immune responses, which are important for diagnosis, are unusually long-lasting, *e.g*., elevated IgM antibodies have been reported to be present 11 years after infection and low, but significant IgG antibody titers detectable in 50% of individuals up to 25 years after infection (Koskela and Salminen, 1985; Ericsson et al., 1994; Maurin, 2020).

The aim of the present study was to determine the kinetics of the IgM and IgG antibody titers up to one year after tularemia and to assess whether determination of individual titers is of diagnostic relevance.

## Materials and methods

### Study population

An outbreak of ulceroglandular tularemia occurred in several parts of Sweden 2019 and a high number of cases were recorded in the county of Hälsingland and in particular in the township of Ljusdal. There is one health center, Ljusdal-Ramsjö hälsocentral, in the township. One of the attending physicians of the health center contacted patients and informed them about the aim of the study and obtained written informed consent. Ethical approvals for the study were received from the Regional Ethical Review Committee, Umeå 2016/335-31 and the Swedish Ethical Review Authority, 2019-01567.

### Patients and serum preparation

The study group included 12 females, 29 - 73 years old, median age of 68 years, and 7 males, 61 - 79 years old, median age 66 years. They were clinically diagnosed with ulceroglandular tularemia within a 2-week period in late July or early August 2019 and the diagnosis was confirmed by the identification of significantly increased IgG and IgM titers. Additional details about the patients are provided in **Table 1**.

**Table 1:** Data regarding the patients included in the study.

Individual no.	Age	Gender	Onset of symptoms	Date of diagnosis
1.	48	Female	28/07/2019	03/08/2019
2.	73	Female	28/07/2019	03/08/2019
3.	68	Male	30/07/2019	02/08/2019
4.	32	Female	01/08/2019	03/08/2019
5.	73	Female	01/08/2019	07/08/2019
6.	28	Female	04/08/2019	08/08/2019
7.	63	Male	24/07/2019	30/07/2019
8.	70	Male	24/07/2019	28/07/2019
9.	60	Male	22/07/2019	30/07/2019
10.	64	Female	02/08/2019	03/08/2019
11.	70	Female	05/08/2019	07/08/2019
12.	78	Male	24/07/2019	01/08/2019
13.	65	Male	27/07/2019	02/08/2019
14.	61	Male	21/07/2019	25/07/2019
15.	52	Female	26/07/2019	01/08/2019
16.	73	Female	29/07/2019	31/07/2019
17.	39	Female	21/07/2019	21/07/2019
18.	72	Female	29/07/2019	30/07/2019
19.	72	Female	19/07/2019	19/07/2019

Venous blood was drawn using BD vacutainer serum tubes (Becton Dickinson, Plymouth, United Kingdom) and serum prepared as described by the manufacturer. Serum samples were obtained at one, two, four, six and 12 months after first sign of illness. The serum was stored at 4°C and analyzed by ELISA within two days after sampling.

### Reagents

Goat anti-human IgM- and IgG-alkaline phosphatase antibodies and NUNC MaxiSorp plates were purchased from Sigma Aldrich, Darmstadt, Germany. Highly purified *F. tularensis* LPS was a generous gift from Dr Wayne Conlan, NRC-CNRC, Ottawa, Canada. The LPS was purified as described (Vinogradov et al., 2002).

### Titration of calibrator serum

A calibrator serum was included in each ELISA plate to normalize data. The serum had been collected from a person vaccinated with *F. tularensis* LVS and tested for antibody titers using two-fold serial dilutions, 100-12,800, and an appropriate dilution was selected as a calibrator serum.

### Measurement of *F. tularensis*-specific IgM and IgG antibody titers

An ELISA was used to determine IgM and IgG antibody titers. The wells of Nunc MaxiSorp plates (Sigma Aldrich) were coated with 100 µl of *F. tularensis* LPS (12 µg/ml) diluted in 0.05 M of carbonate-bicarbonate, pH 9.5 and incubated overnight at 26°C. The antigen was removed and wells filled with PBS + BSA 0.5% and stored at -80°C until use. Uncoated wells were included to control for non-specific binding. Before addition of serum samples, the plates were washed four times with PBS + 0.05% BSA. The patient sera were diluted 1,000-fold in incubation buffer (PBS + 0.05% of Tween-20). One hundred µl of the diluted serum was added to coated and uncoated wells. The plates were incubated at 26°C for 3 h and thereafter washed. The anti-IgG- and anti-IgM-conjugated alkaline phosphatase antibodies were diluted 5,000-fold in incubation buffer and 100 µl added to each well. After overnight incubation at 26°C, plates were washed three times before 100 µl of alkaline phosphatase substrate was added to each well. The reaction was stopped by adding 50 µl of 3.0 M of NaOH per well when wells incubated with the calibrator serum had reached an OD of 1.0 A_405_. The relative titer of each serum sample was calculated by subtracting the value of the non-coated wells and thereafter multiplying the value with the/correction factor and the dilution factor. The correction factor was calculated by dividing the nominal value of the calibrator serum, 1.0, with the obtained value of wells with the calibrator serum. Based on an extensive collection of sera from donors with no known exposure to *F. tularensis*, 100 - 200 was considered to be a threshold value and a value of > 200 as positive.

Since 2010, approximately 14,850 serum samples had been analyzed by the method. Of these, 1,801 were IgM positive and 2,520 IgG positive. Based on these results, it was determined that the cut-off of 100 had a sensitivity of 100% for both IgM and IgG and a specificity of 93.6% for IgG and 97.3% for IgM. The PPV for IgG was 97.0% and for IgM 99.6% and the NPV for both 100%. For the cut-off of 200, the sensitivity was 97.3% for IgG and 98.3% for IgM. The specificity was in both instances 100.0%. The PPV for both were 100% and the NPV was 99.5% for IgG and 99.8% for IgM. The intra-assay CV was 4.6% and the inter-assay CV 9.6%.

### Statistical analysis

Student`s *t*-test were used. A *P* value of < 0.05 was considered significant.

## Results

### Kinetic responses of *F. tularensis*-specific antibodies

**Figure 1:**
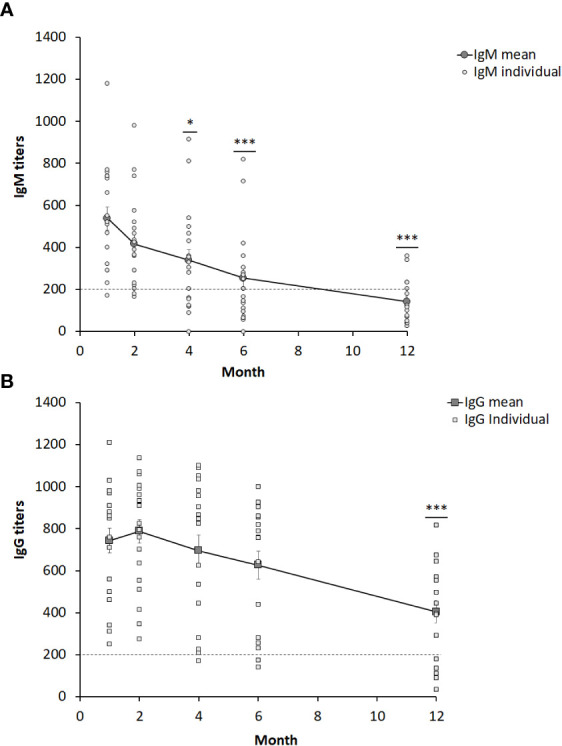
IgM **(A)** and IgG **(B)** serum titers from 19 individuals diagnosed with ulceroglandular tularemia. The dotted line indicates the cut-off value of 200. Stars indicate significant differences compared to the one-month value (*P < 0.05, ***P < 0.001).

**Figure 2:**
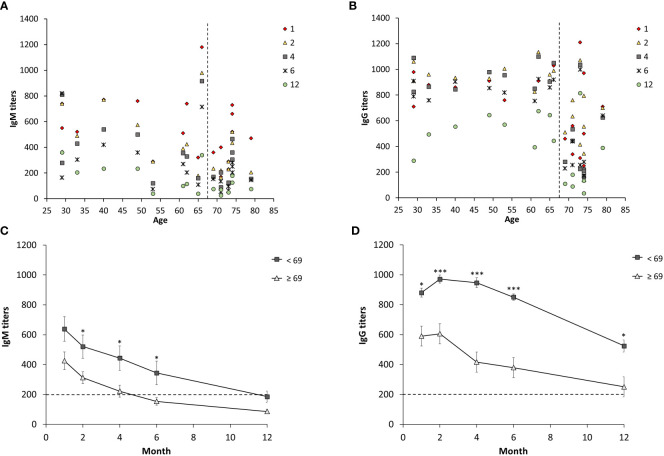
Age-dependent IgM and IgG serum titers from 19 individuals diagnosed with ulceroglandular tularemia. Diagram **(A, B)** show individual IgM and IgG titers respectively. Diagram **(C)** and **(D)** show mean values ± SEM of IgM and IgG, respectively, in individuals < 69 years (n = 10) and ≥ 69 years (n = 9) at a given time point. Stars indicate significant differences between the two age groups at a given time point (*p < 0.05, ***p < 0.001).

Sera from 19 individuals with ulceroglandular tularemia were obtained on 5 occasions, 1 -12 months, after onset of disease and analyzed for the presence of IgM and IgG antibodies. Peak average antibody levels were observed at the one-month time point for IgM, 537 ± 54, and at the two-month time point for IgG, 789 ± 56 (**Figure 1**). All individual IgG and all but one of the IgM values at these time points were above the cut-off (**Figure 1**). Both IgG and IgM antibody levels declined linearly thereafter with rather similar kinetics. Despite the decline, the mean IgG titers were not significantly lower compared to 1-month before the 12-month time point and the mean IgM titers were not significantly lower before the 4-month time point. Notably, both the values at the 12-month time point, 404 ± 52 (IgG), and 4-month, 339 ± 50 (IgM), were significantly above the cut-off of the assay, 200. The only value below the cut-off was the 12-month IgM value, 142 ± 23, but still a threshold value. Mean IgG or IgM values were not significantly different between male and females irrespective of time point analyzed (not shown). To further analyze if there were any gender differences, only individuals older than 60 years were compared, 7 females 65 – 74 years old and 7 males 61 – 79 years old, but, again, no IgG or IgM titer differences at any time point were found. Analysis of age-related titers revealed a decline of titers among elderly persons (**Figures 2A, B**). Based on this observation, titers were analyzed for groups of individuals < 69 years or ≥ 69 years old (**Figures 2C, D**). It was observed that the mean values were higher for the group < 69 years old and the differences were significant for the 2, 4, and 6-month time points for IgM and for all five time points for IgG (**Figures 2C, D**).

## Discussion

Specific knowledge regarding the kinetics of humoral immune responses after infection is of much relevance from a clinical standpoint, since the information can be utilized to pinpoint when infection occurred and therefore be diagnostically important. Serological responses to many infections are characterized by IgM titers peaking within a couple of weeks and thereafter rather rapidly declining, sometimes disappearing within weeks or a few months, whereas IgG titers peak more slowly, but persist for much longer, at least many months (Vainionpää et al., 2015). Serological diagnosis of an acute infection is often based on the demonstration of high levels of IgM antibodies, or a significant increase in the levels of antibodies between two consecutive samples taken one to four weeks apart.

The use of serology for diagnosis of tularemia is well established and there are numerous studies demonstrating its utility, reviewed by Maurin (Maurin, 2020). A number of microagglutination tests (MAT) or ELISA tests have been evaluated and they generally demonstrate acceptable sensitivity of 85 - 95% and specificity of 90 - 95%. However, considering that tularemia is a rare disease in many countries, such specificities may still yield an unacceptable high false positive rate. An advantage of ELISA-based tests vs. MAT is that the former detects antibodies about a week earlier than the latter method (Maurin, 2020). The kinetics of the early humoral immune response after tularemia has been studied and, generally, few patients demonstrated significant antibody levels during the first week; 14% were found to be positive using ELISA, whereas 67% were positive during the second week, and 100% during the third and fourth weeks (Yanes et al., 2018). Also, other studies have observed that > 90% of the patients demonstrate significant IgG titers within 3-4 weeks (Carlsson et al., 1979; Koskela and Salminen, 1985; Syrjälä et al., 1986; Kilic et al., 2012). The results of the present study agree with the previous studies since we observed peak IgM levels one-month post infection and peak IgG antibodies two months post infection. Moreover, all of the patients seroconverted and demonstrated significantly elevated IgM and IgG titers. Notably, there are no consistent differences between the kinetics of the IgM and IgG antibody responses after tularemia, reviewed by Maurin (Maurin, 2020), in contrast to the serological responses to most other infections when IgG antibody responses remain elevated for much longer than do IgM antibody responses (Vainionpää et al., 2015).

It is well documented that cell-mediated and humoral immune responses after tularemia are very long-lived. In fact, cell-mediated immunity may be life-long since > 95% of individuals were found to present with significant cell-mediated immune responses 25 years after tularemia (Ericsson et al., 1994). At that time, humoral immunity had waned, but notably, 50% of the individuals still demonstrated IgG titers above cut-off, or threshold values (Ericsson et al., 1994). A Norwegian study demonstrated that 95% of individuals showed significant IgG titers 8 years after infection and a Finnish study that 100% showed significant IgM antibody levels 11 years after infection (Koskela and Salminen, 1985; Bevanger et al., 1994). Persistence of specific immune responses may be due to repeated exposure, however, in view of the low incidence of tularemia in the specific regions, this is an unlikely reason for the aforementioned long-lived immune persistence. The persistence of elevated IgG antibodies for decades is not unprecedented, however, since antibody responses after viral infections have been found to have half-lives of 50 years or more (Amanna et al., 2007). In support of the long-lived immune responses to *F. tularensis*, also in individuals vaccinated with the live vaccine strain of *F. tularensis*, cell-mediated immunity was found to persist for three decades without evidence of decline (Eneslätt et al., 2011).

The present study provides additional information regarding the kinetics after tularemia and demonstrated that there were elevated IgG and IgM antibody titers for at least 12 months after infection. Although the titers waned over time, compared to the average one-month IgG titer, a significant decrease for the group was not observed until the 12-month time point. Compared to the one-month IgM titer, there was significant decrease at the 4-month time point. Notably, the ≥ 69 year-group showed significantly lower antibody levels, a finding not reported previously. This is in agreement with the impaired B-cell responses occurring during ageing and concomitant increased susceptibility to various bacterial and viral infections among elderly (Frasca et al., 2011; Crooke et al., 2019; Palacios-Pedrero et al., 2021). A limitation of the study was the relatively few individuals included, however, the power was still sufficient to identify the age-related decline. Other, more subtle differences may be possible to identify if a larger cohort is analyzed.

Collectively, the data demonstrate long-term persistence of IgM and IgG antibody titers, both of which are significantly elevated during a period of 12 months. From a clinical perspective, this means that low-levels antibody titers are not indicative of acute or recent tularemia. Thus, serological diagnosis has to rely on the demonstration of significant increase of the antibody levels between two samples taken one to four weeks apart, concomitantly with a clinical presentation compatible with tularemia. Other authors have also made the same conclusions (Syrjälä et al., 1986; Bevanger et al., 1994; Yanes et al., 2018).

## Data availability statement

The original contributions presented in the study are included in the article/supplementary material. Further inquiries can be directed to the corresponding author.

## Ethics statement

The studies involving human participants were reviewed and approved by the Regional Ethical Review Committee, Umeå 2016/335-31 and the Swedish Ethical Review Authority, 2019-01567. The patients/participants provided their written informed consent to participate in this study.

## Author contributions

HL and KE performed the experiments. AS, JE, and HL designed the study. HL and AS analyzed the data and wrote the manuscript. HL, JE, KE, and AS reviewed the manuscript. All authors contributed to the article and approved the submitted version.

## References

[B1] Abdellahoum Z. Maurin M. Bitam I. (2020). Tularemia as a mosquito-borne disease. Microorganisms 9(1), 26. doi: 10.3390/microorganisms9010026 33374861 PMC7823759

[B2] Amanna I. J. Carlson N. E. Slifka M. K. (2007). Duration of humoral immunity to common viral and vaccine antigens. N Engl. J. Med. 357, 1903–1915. doi: 10.1056/NEJMoa066092 17989383

[B3] Anda P. Pearson A. Tärnvik A. (2007). “Clinical expression in humans,” in WHO guidelines: Tularemia (World Health Organization, Geneva, Switzerland: WHO press), 11–19.

[B4] Bevanger L. Maeland J. A. Kvan A. I. (1994). Comparative analysis of antibodies to *Francisella tularensis* antigens during the acute phase of tularemia and eight years later. Clin. Diagn. Lab. Immunol. 1, 238–240. doi: 10.1128/cdli.1.2.238-240.1994 7496953 PMC368235

[B5] Carlsson H. E. Lindberg A. A. Lindberg G. Hederstedt B. Karlsson K. A. Agell B. O. (1979). Enzyme-linked immunosorbent assay for immunological diagnosis of human tularemia. J. Clin. Microbiol. 10, 615–621. doi: 10.1128/jcm.10.5.615-621.1979 120873 PMC273233

[B6] Conlan J. W. (2011). Tularemia vaccines: recent developments and remaining hurdles. Future Microbiol. 6, 391–405. doi: 10.2217/fmb.11.22 21526941

[B7] Crooke S. N. Ovsyannikova I. G. Poland G. A. Kennedy R. B. (2019). Immunosenescence and human vaccine immune responses. Immun. Ageing 16, 25. doi: 10.1186/s12979-019-0164-9 31528180 PMC6743147

[B8] Elkins K. L. Kurtz S. L. De Pascalis R. (2016). Progress, challenges, and opportunities in *Francisella* vaccine development. Expert Rev. Vaccines 15, 1183–1196. doi: 10.1586/14760584.2016.1170601 27010448

[B9] Eneslätt K. Rietz C. Ryden P. Stöven S. House R. V. Wolfraim L. A.. (2011). Persistence of cell-mediated immunity three decades after vaccination with the live vaccine strain of *Francisella tularensis* . Eur. J. Immunol. 41, 974–980. doi: 10.1002/eji.201040923 21442618 PMC3516913

[B10] Ericsson M. Kroca M. Johansson T. Sjöstedt A. Tärnvik A. (2001). Long-lasting recall response of CD4+ and CD8+ alphabeta T cells, but not gammadelta T cells, to heat shock proteins of *Francisella tularensis* . Scand. J. Infect. Dis. 33, 145–152. doi: 10.1080/003655401750065562 11233852

[B11] Ericsson M. Sandstrom G. Sjöstedt A. Tärnvik A. (1994). Persistence of cell-mediated immunity and decline of humoral immunity to the intracellular bacterium *Francisella tularensis* 25 years after natural infection. J. Infect. Dis. 170, 110–114. doi: 10.1093/infdis/170.1.110 8014484

[B12] Frasca D. Diaz A. Romero M. Landin A. M. Blomberg B. B. (2011). Age effects on b cells and humoral immunity in humans. Ageing Res. Rev. 10, 330–335. doi: 10.1016/j.arr.2010.08.004 20728581 PMC3040253

[B13] Hestvik G. Warns-Petit E. Smith L. A. Fox N. J. Uhlhorn H. Artois M.. (2015). The status of tularemia in Europe in a one-health context: a review. Epidemiol. Infect. 143, 2137–2160. doi: 10.1017/S0950268814002398 25266682 PMC9506979

[B14] Kilic S. Celebi B. Yesilyurt M. (2012). Evaluation of a commercial immunochromatographic assay for the serologic diagnosis of tularemia. Diagn. Microbiol. Infect. Dis. 74, 1–5. doi: 10.1016/j.diagmicrobio.2012.05.030 22770772

[B15] Kirimanjeswara G. S. Olmos S. Bakshi C. S. Metzger D. W. (2008). Humoral and cell-mediated immunity to the intracellular pathogen *Francisella tularensis* . Immunol. Rev. 225, 244–255. doi: 10.1111/j.1600-065X.2008.00689.x 18837786 PMC4871322

[B16] Koskela P. Salminen A. (1985). Humoral immunity against *Francisella tularensis* after natural infection. J. Clin. Microbiol. 22, 973–979. doi: 10.1128/jcm.22.6.973-979.1985 4066925 PMC271862

[B17] Maurin M. (2020). *Francisella tularensis*, tularemia and serological diagnosis. Front. Cell Infect. Microbiol. 10. doi: 10.3389/fcimb.2020.512090 PMC7649319 33194778

[B18] Palacios-Pedrero M. A. Osterhaus A. Becker T. Elbahesh H. Rimmelzwaan G. F. Saletti G. (2021). Aging and options to halt declining immunity to virus infections. Front. Immunol. 12. doi: 10.3389/fimmu.2021.681449 PMC8149791 34054872

[B19] Petersen J. M. Dennis D. T. Beard C. B. (2017). “Tularemia,” in Infectious diseases, 4th ed. Eds. Jonathan Cohen W. G. P. Opal S. M. (Elsevier), 1085–1090. doi: 10.1016/B978-0-7020-6285-8.00127-1

[B20] Rossow H. Ollgren J. Hytonen J. Rissanen H. Huitu O. Henttonen H.. (2015). Incidence and seroprevalence of tularaemia in Finland 1995 To 2013: regional epidemics with cyclic pattern. Euro Surveill 20, 21209. doi: 10.2807/1560-7917.es2015.20.33.21209 26314404

[B21] Sjöstedt A. (2007). Tularemia: history, epidemiology, pathogen physiology, and clinical manifestations. Ann. N Y Acad. Sci. 1105, 1–29. doi: 10.1196/annals.1409.009 17395726

[B22] Syrjälä H. Koskela P. Ripatti T. Salminen A. Herva E. (1986). Agglutination and ELISA methods in the diagnosis of tularemia in different clinical forms and severities of the disease. J. Infect. Dis. 153, 142–145. doi: 10.1093/infdis/153.1.142 3941279

[B23] Tärnvik A. Berglund L. (2003). Tularaemia. Eur. Respir. J. 21, 361–373. doi: 10.1183/09031936.03.00088903 12608453

[B24] Tärnvik A. Priebe H. S. Grunow R. (2004). Tularaemia in Europe: an epidemiological overview. Scand. J. Infect. Dis. 36, 350–355. doi: 10.1080/00365540410020442 15287379

[B25] Tärnvik A. Sandström G. Sjöstedt A. (1996). Epidemiological analysis of tularemia in Sweden 1931-1993. FEMS Immunol. Med. Microbiol. 13, 201–204. doi: 10.1111/j.1574-695X.1996.tb00237.x 8861029

[B26] Telford S. R. 3rd Goethert H. K. (2020). Ecology of *Francisella tularensis* . Annu. Rev. Entomol 65, 351–372. doi: 10.1146/annurev-ento-011019-025134 31600457 PMC8300880

[B27] Vainionpää R. Waris M. Leinikki P. (2015). Diagnostic techniques: Serological and molecular approaches. Reference Module Biomed. Sci. doi: 10.1016/B978-0-12-801238-3.02558-7

[B28] Vinogradov E. Perry M. B. Conlan J. W. (2002). Structural analysis of *Francisella tularensis* lipopolysaccharide. Eur. J. Biochem. 269, 6112–6118. doi: 10.1046/j.1432-1033.2002.03321.x 12473106

[B29] Yanes H. Hennebique A. Pelloux I. Boisset S. Bicout D. J. Caspar Y.. (2018). Evaluation of in-house and commercial serological tests for diagnosis of human tularemia. J. Clin. Microbiol. 56(1):e01440–17. doi: 10.1128/JCM.01440-17 PMC5744216 29118164

